# Correct vaccination practices begin with proper education: The effect of professional training on vaccine advocacy among healthcare professionals

**DOI:** 10.7705/biomedica.8136

**Published:** 2026-08-20

**Authors:** Öznur Tiryaki

**Affiliations:** 1 Departmant of Midwifery, Faculty of Health Science, Sakarya University, Sakarya, Türkiye Sakarya University Departmant of Midwifery Faculty of Health Science Sakarya University Sakarya Turkey

**Keywords:** Nurses, midwifery, vaccines, health promotion, enfermeras y enfermeros, partería, vacunas, promoción de la salud

## Abstract

**Introduction.:**

Healthcare professionals, particularly midwives and nurses, have played a fundamental role in global immunization efforts by acting as the primary providers responsible for vaccine administration.

**Objective.:**

This study aims to determine whether there is a statistically significant difference in Childhood Vaccination Advocacy Scale scores before and after vaccine advocacy awareness training.

**Materials and methods.:**

Data were collected using a questionnaire on demographic characteristics and knowledge regarding vaccine administration, developed by the researcher in accordance with the literature, as well as the Vaccine Advocacy Scale for Childhood Vaccines. This research was designed as a single-group, pre-test and post-test quasi-experimental study. Pre-test and post-test scores were compared using the Wilcoxon signed-rank test. A significance level of p < 0.05 was considered statistically significant.

**Results.:**

The study was conducted with 249 midwives and/or nurses. The mean age of the participants was 39.40 ± 7.43 years, with an average of 18.01 ± 7.99 years of professional experience. The mean total score on the Vaccine Advocacy Scale for Childhood Vaccines was 45.19 ± 2.85 before the training and 46.95 ± 1.99 after the training. A Wilcoxon signed-rank test revealed that the increase in post-training scores was statistically significant (p = 0.000).

**Conclusion.:**

The findings indicate that participants had relatively high levels of vaccine advocacy even prior to the training. However, the statistically significant improvement in scores post-training clearly demonstrates the effectiveness of the educational intervention.

## Resumen

**Introducción.:**

Los profesionales de la salud, especialmente las matronas y las enfermeras, han desempeñado un papel fundamental en los esfuerzos de inmunización a nivel mundial al ser los principales responsables de la administración de vacunas.

**Objetivo.:**

Determinar si existe una diferencia estadísticamente significativa en las puntuaciones de la *Childhood Vaccination Advocacy Scale* antes y después de la capacitación a enfermeras y matronas sobre la concienciación de la promoción de la vacunación.

**Materiales y métodos.:**

Los datos se obtuvieron mediante un cuestionario de características demográficas y conocimientos sobre vacunación, elaborado por la investigadora con base en la literatura, y mediante la *Childhood Vaccination Advocacy Scale.* El estudio se diseñó como un estudio cuasiexperimental de un solo grupo con preprueba y posprueba. Las puntuaciones de la preprueba y la posprueba se compararon mediante la prueba de rangos con signo de Wilcoxon.

**Resultados.:**

El estudio se realizó con 249 enfermeras y matronas, quienes tenían una edad media de 39,40 ± 7,43 años, con una experiencia profesional promedio de 18,01 ± 7,99 años. La puntuación media total en la *Childhood Vaccination Advocacy Scale* fue de 45,19 ± 2,85 antes de la formación y de 46,95 ± 1,99 después de ella. La prueba de Wilcoxon reveló que el aumento de las puntuaciones posteriores a la formación era estadísticamente significativo (p = 0,000).

**Conclusión.:**

Los resultados indican que las participantes ya tenían niveles relativamente altos para la promoción de las vacunas antes de la capacitación. Sin embargo, la mejora estadísticamente significativa de las puntuaciones después de la capacitación demuestra la eficacia de la intervención educativa.

Vaccination is a fundamental responsibility of primary healthcare and is recognized as a human right. According to vaccination programs, vaccines are administered against more than twenty diseases, helping individuals of all ages to live longer and healthier lives. Within the framework of the Immunization Agenda 2030, it is anticipated that between 2021 and 2030, everyone, everywhere, and at every age will fully benefit from vaccines to improve health and well-being ^1^. However, the success of immunization programs depends on the acceptance of vaccination and the achievement of high vaccination coverage within the community ^2^. Vaccine hesitancy, which is considered one of the top ten threats to global health, is regarded as a critical barrier to maximizing immunization rates ^3^.

Addressing vaccine hesitancy or refusal, and particularly improving vaccination rates among young people, requires enhancing knowledge, attitudes, and beliefs related to immunization ^4^. Vaccine literacy, as an extension of health literacy, refers to the degree to which individuals can obtain, process, and understand basic vaccine information and services necessary to make appropriate vaccination decisions ^5^. Increasing vaccine literacy is increasingly recognized as a crucial strategy in combating vaccine hesitancy and promoting informed decision-making regarding vaccination ^4^. Healthcare professionals play a vital role in this context, often serving as the most trusted sources of information during the vaccination decision-making process. Their knowledge, attitudes, and communication skills regarding vaccination significantly influence their own vaccination behaviors, vaccine recommendations, and patients' vaccination decisions 6-8.

Healthcare professionals, particularly midwives and nurses, play a pivotal role in global immunization efforts as the primary groups responsible for administering vaccines. Additionally, midwives and nurses hold significant roles in positively influencing public opinion and building trust toward preventive health services. Healthcare workers who receive their own vaccinations are more likely to recommend and facilitate vaccination within the community ^9^. Moreover, since healthcare workers often come into contact with vulnerable populations, especially children, they face a higher risk of transmitting infections that could lead to more severe outcomes; therefore, it is critically important for them to be vaccinated themselves ^10^. The attitudes of healthcare professionals towards vaccines can influence whether they recommend vaccinations to patients. Sometimes, healthcare workers require up-to-date information on new vaccines, including their benefits, administration methods, and dosages ^11^.

The role of healthcare professionals in increasing vaccine acceptance in the community extends beyond providing technical knowledge; it also encompasses multidimensional responsibilities such as building trust, managing concerns, and effective communication. Therefore, healthcare workers need to be supported with comprehensive training programs to effectively fulfill their roles as vaccine advocates. Such training aims to enhance healthcare professionals' vaccine advocacy and cultural competency skills. By emphasizing intercultural communication, empowerment, and advocacy, this curriculum ensures that healthcare workers can deliver effective vaccine communication across diverse cultural contexts. This approach demonstrates that equipping healthcare professionals with knowledge, skills, and resources is critical to protecting community health and developing sustainable healthcare systems ^12^^-^^15^.

Vaccination in children represents one of the most common and important applications of injections. Vaccines are vital for strengthening children's immune systems, preventing infectious diseases, and protecting public health. Therefore, administering vaccines to children with correct techniques, in hygienic conditions, and appropriate for their age is of utmost importance ^16^.

The aim of this study was to evaluate the effect of a vaccine advocacy awareness training program on vaccine advocacy levels among midwives and nurses using a single-group pre-test and post-test design. Specifically, the study aims to determine whether participation in an in-service vaccine training program leads to a significant improvement in vaccine advocacy attitudes and practices among healthcare professionals, thereby supporting vaccine acceptance in the community.

## Research questions

- What are the demographic and professional characteristics of midwives and nurses participating in the study?
- What are the baseline Vaccine Advocacy Scale for Childhood Vaccines levels of midwives and nurses before the training?
- Is there a statistically significant difference in Vaccine Advocacy Scale for Childhood Vaccines scores before and after the vaccine advocacy awareness training?

## Materials and method

### Type of study

This research was designed as a single-group, pre-test and post-test quasi-experimental study.

### Study setting and duration

The study was conducted between May 1st. and May 31st., 2025, among midwives and nurses working at a primary healthcare facility in a province located in the western region of Turkey.

### Population and sample

The population consisted of 312 midwives and nurses who participated in the *Safe Vaccine Practices in Pediatrics* training organized by the Provincial Health Directorate during the data collection period.

The study sample included 249 volunteer midwives and nurses recruited using a non-probabilistic, self-selected sampling method. An a priori power analysis was conducted using G*Power, version 3.1.9.7, for a single-group, pretest and post-test (quasi-experimental) design. Based on a medium effect size (dz = 0.50), a two-tailed significance level of α = 0.05, and a statistical power of 80%, the minimum required sample size was calculated as 34 participants.

This study was designed, conducted, and reported in accordance with the Joanna Briggs Institute Critical Appraisal Checklist for Quasi-Experimental Studies.

### Inclusion criteria

Being a midwife or nurse, attending the planned training, working in a primary healthcare facility, and voluntarily agreeing to participate in the study.

### Exclusion criteria

Participants were excluded if they did not complete the training program in its entirety, had incomplete or missing pre-test or post-test data, were not actively involved in vaccination-related services during the study period, or withdrew from the study before post-intervention assessment.

### Data collection

Data were collected by the researcher using a demographic and vaccine administration knowledge questionnaire, along with the Vaccine Advocacy Scale. The demographic and knowledge questionnaire was developed based on the literature ^17^^,^^18^, and included questions on participants' profession, age, education level, total years of professional experience, duration of work in primary care, and parameters to consider during vaccine administration (e.g., child's age, weight, needle gauge and length, injection site, etc.).

### Vaccine advocacy scale for childhood vaccines

This scale was developed to assess adults' advocacy behaviors toward childhood vaccinations. It consists of 12 items structured on a 4-point Likert scale. There are no reverse-coded items in the scale. Items 1, 2, 3, 4, 5, 6, 7, and 12 constitute the "individual development" subdimension, while items 8, 9, 10, and 11 comprise the "social advocacy" subdimension. The total score ranges from 12 to 48, with no cut-off point. Higher total scores are interpreted as higher levels of vaccine advocacy behavior ^19^. The Cronbach's alpha value of the original scale was reported as 0.92; in the current study, it was calculated as 0.663 for the pre-test and 0.765 for the post-test.

### Content of the training on safe vaccination practices in pediatrics

All midwives and nurses working at family health centers in the city where the study was conducted were divided into three groups. Each group was invited to the training session on separate dates. The same training presentation was delivered by the same instructor for all three groups. The instructor holds the academic title of Associate Professor in Pediatric Nursing and has 21 years of experience in pediatric nursing. The duration of each training session was planned as 120 minutes. The training content was developed by the researcher based on the literature ^17^^,^^20^^-^^22^, and professional experience.

The presentation included information on safe zones used in vaccination and intramuscular (IM) injection, and emphasized the importance of weight, age, and muscle mass in selecting the injection site. The influence of needle size and medication volume on site selection was discussed. Non-pharmacological methods for managing pain and fear during vaccination and IM injection procedures were also explained. The global protective effect of vaccines and the importance of strengthening healthcare professionals' roles in vaccine advocacy and effective communication were addressed. In the final 20 minutes of the session, a role-play on vaccine refusal was conducted to conclude the training.

### Data analysis

Statistical analyses of the data were conducted using the Statistical Package for the Social Sciences™ (SPSS), version 26.0, for Windows. Descriptive statistics were presented as arithmetic means and standard deviations for numerically measured variables. The Kolmogorov-Smirnov test was used to assess the normality of the scale distribution. Based on skewness and kurtosis values, the data were determined not to follow a normal distribution. As the data did not meet the assumption of normality, non-parametric analyses were employed. Pre-test and post-test scores were compared using the Wilcoxon signed-rank test. A significance level of p < 0.05 was considered statistically significant.

### Ethical considerations

Permission to use the scale was obtained via email from the corresponding authors. Prior to data collection, ethical approval was obtained from the Sakarya University Ethics Committee for Social and Human Sciences (April 24, 2025 - Reference No: 84/75). Before the training sessions, participants were informed about the study, and written and verbal informed consent was obtained from those who agreed to participate. In accordance with ethical standards, the principles of the Declaration of Helsinki were followed, and data confidentiality and information security were ensured throughout the study.

### Results

Among the 249 participants in the study, 54.2% held the title of midwife and 45.8% were nurses. The mean age of the midwives and nurses was 39.40 ± 7.43 years, and their average duration of professional experience was 18.01 ± 7.99 years. It was found that 47.8% had been working in family health centers for 11 years or more. All participants had administered at least one optional (paid) vaccine, with the rotavirus vaccine being the most frequently administered (59.4%).

When evaluating their proficiency in implementing the national immunization schedule, 78.3% of the participants rated themselves as proficient. It was also determined that 9% had previously experienced incorrect vaccine administration, and 99.6% had encountered vaccine refusal. Furthermore, 98% of participants reported fully vaccinating their own children according to the national immunization schedule, while 75.5% had administered optional vaccines to their children. Notably, 50.6% expressed the desire for the meningococcal vaccine to be offered free of charge (table 1).

**Table 1:** Demographic characteristics and vaccination experience of participants (N = 249)

Variable		n	%	Variable		n	%
Profession				Education status			
	Midwife	135	54.2		High school/Associate degree	85	34.1
	Nurse	114	45.8		Bachelor's degree	152	61.0
Years of experience in primary health center					Postgraduate		4.8
	< 1	36	14.5	Success in applying the national vaccination schedule			
	1-5	65	26.1				
	6-10	29	11.6		Good	195	78.3
	>11	119	47.8		Moderate	54	21.7
Encounter with post-vaccination side effects						Poor	0
	Yes	80	32.1	Experience with incorrect vaccination			
	No	169	67.9		Yes	9	3.6
Experience administering paid vaccines					No		96.4
	Yes	249	100	Most frequently administered paid vaccine			
	No	0	0				
Vaccine you wish to be free of charge					Meningococcal	101	40.6
	Meningococcal	126	50.6		Rotavirus	148	59,4
	Rotavirus	61	24.5	Situations involving vaccine refusal			
	HPV	8	3.2		Yes	248	99.6
	All of them	54	21.7		No	1	0.4
Applying national vaccination schedule to own child				Applying special/paid vaccine to own child			
	Yes	244	98.0		Yes	188	75.5
	No	5	2.0		No	61	24.5
Injection site preferences by age group (0-3 years, vaccine site)				Injection site preferences by age group (0-3 years, IM injection site)			
	*Rectus femoris*	48	19.3		*Rectus femoris*	41	16.5
	*Vastus lateralis*	158	63.5		*Vastus lateralis*	176	70.7
	Ventrogluteal	9	3.6		Ventrogluteal	4	1.6
	Dorsogluteal	2	0.8		Dorsogluteal	5	2.0
	Deltoid	32	12.9		Deltoid	23	9.2
Injection site preferences by age group (0-3 years, vaccine site)				Injection site preferences by age group (3-18 years, IMI site)			
	*Rectus femoris*	5	2.0		*Rectus femoris*	19	7.6
	*Vastus lateralis*	14	5.6		*Vastus lateralis*	90	36.1
	Ventrogluteal	4	1.6		Ventrogluteal	43	17.3
	Dorsogluteal	7	2.8		Dorsogluteal	96	38.6
	Deltoid	219	88.0		Deltoid	1	0.4
Other variables		Mean ± SD					
Age		39.40 ± 7.43 (min. 27, max. 61)					
Total years in profession		18.01 ± 7.99 (min. 2, max. 38)					

HPV: Human papillomavirus; IM: Intramuscular

When participants were asked about their knowledge regarding the preferred sites for vaccine and IM injection administration, it was found that in the 0-3 age group, 63.5% of vaccines and 70.7% of IM injections were administered in the *vastus lateralis* muscle. In the 3-18 age group, 88% of vaccines were administered in the deltoid region, while 38.6% of IM injections were administered in the dorsogluteal region and 36.1% in the *vastus lateralis* (table 1).

The mean total score of the Vaccine Advocacy Scale for Childhood Vaccines before the training was 45.19 ± 2.85, and after the training, it increased to 46.95 ± 1.99. According to the Wilcoxon signed-rank test, the increase in the post-training mean score was statistically significant (p = 0.000). The predominance of positive ranks indicates a general increase in post-test scores (table 2).

**Table 2:** Comparison of pre- and post-education total scores of the vaccine advocacy scale (N = 249)

Rank	n	Mean rank	Sum rank	z*	p**
Negative ranks (post-test < pre-test)	22	65.16	1433.50	-9.587	0.000
Positive ranks (post-test > pre-test)	157	93.48	14676.50		
Ties (post-test = pre-test)	70	----	----		

* z: Wilcoxon signed-rank test

**p < 0.05 (statistically significant)

When comparing the item-specific mean scores of the scale before and after the training, the most significant increases were observed in items 3, 7, and 12. These items respectively state: "I read reports about childhood vaccines from official sources such as the World Health Organization and the Ministry of Health," "I can recommend reliable information sources to parents regarding childhood vaccines," and "I follow/read scientific information about childhood vaccines" (table 3).

**Table 3:** Comparative analysis of item-level pre-test and post-test scores of the vaccine advocacy scale (N = 249)

	Min	Max	Mean	SD	Min	Max	Mean	SD
	(pre)	(pre)	(pre)	(pre)	(post)	(post)	(post)	(post)
Item 1	2.00	4.00	3.9237	0.28077	2.00	4.00	3.9036	0.34598
Item 2	1.00	4.00	3.7871	0.50688	1.00	4.00	3.8715	0.41098
Item 3	1.00	4.00	**3.4096**	0.77828	1.00	4.00	**3.7952**	0.47754
Item 4	1.00	4.00	3.7871	0.50688	2.00	4.00	3.9076	0.31671
Item 5	1.00	4.00	3.8514	0.41882	3.00	4.00	3.9639	0.18703
Item 6	2.00	4.00	3.8876	0.32905	3.00	4.00	3.9639	0.18703
Item 7	1.00	4.00	**3.5221**	0.69016	1.00	4.00	**3.8233**	0.45002
Item 8	3.00	4.00	3.9880	0.10932	3.00	4.00	3.9839	0.12598
Item 9	1.00	4.00	3.7631	0.51985	2.00	4.00	3.9197	0.28676
Item 10	1.00	4.00	3.7791	0.67457	3.00	4.00	3.9719	0.16563
Item 11	3.00	4.00	3.9357	0.24570	3.00	4.00	3.9759	0.15366
Item 12	1.00	4.00	**3.5582**	0.65809	2.00	4.00	**3.8755**	0.38699

Additionally, all participants reported paying attention to the needle length of the syringe they would use and considering the child's age in the selection of the injection site. Furthermore, 90.4% of midwives and nurses reported inquiring about the child's weight, 95.2% performed aspiration during injections, and 91.2% employed at least one non-pharmacological method (e.g., breastfeeding, kangaroo care, loose swaddling, colorful toys with sounds, massage) to manage pain. Finally, 57% stated that a 2 cm distance between two injections administered in the same leg was sufficient.

## Discussion

Midwives and nurses are fundamental healthcare professionals who play a crucial role in patient care and fulfill many essential responsibilities. Among these, medication administration is one of the most critical duties. Injection practices are particularly significant in ensuring the effective progress of treatment processes ^23^. Performing these procedures with correct techniques and under hygienic conditions ensures the effectiveness of the medication and helps prevent potential complications ^24^. Mastery of injection techniques is essential for ensuring patient safety. These professionals must apply the correct techniques for each type of injection, ensure the sterility of the equipment used, and prioritize patient safety at every step ^25^.

Furthermore, administering injections in children requires specialized knowledge and skills. The anatomical and physiological characteristics of children influence both injection techniques and the selection of equipment ^26^. A previous study reported that the ventrogluteal site is sufficiently developed even in infants aged 1-12 months, and that in children aged 12-36 months, the ventrogluteal site was found to be thicker than the *vastus lateralis* site ^27^. In this study, participants reported using the *vastus lateralis* site for children under three years of age, and for older children, the deltoid region was preferred for vaccinations, while both the *vastus lateralis* and dorsogluteal regions were used for IM injections. As emphasized in the literature, the choice of injection site and appropriate needle length is of vital importance. In-service training programs should be implemented for nurses and midwives after graduation to ensure they have access to up-to-date knowledge. Educational interventions such as the one in this study can promote the use of the ventrogluteal site, thereby enhancing both patient and healthcare worker safety.

Participants reported that they generally used non-pharmacological pain management methods during injection procedures. In newborns and infants, breastfeeding, oral sucrose, and non-nutritive sucking are recommended to reduce vaccine-related pain ^28^. Breastfeeding has been found to be effective in reducing pain during intramuscular vaccination in infants aged between one and four months ^29^. Another study reported that distraction and breastfeeding were effective in reducing post-vaccination pain in infants aged 1-12 months ^30^. It is also known that holding the child in a sitting position, applying pressure to the injection site, and administering the injection quickly are effective strategies for pain reduction ^31^.

Low-cost and easy-to-use tools such as the ShotBlocker and Brazil's Pikluc, as well as the BUZZY™ device, which provides vibration, have been shown to reduce injection-related pain ^32^^,^^33^. Especially in children aged 3-6 years, local cooling (ice) applied to the vaccination site is emphasized as a safe and effective method ^34^. The CARD system (comfort, ask, relax, distract), used during school-age vaccination programs, is noted to reduce stress in children and promote effective communication with healthcare providers ^35^. The use of virtual reality headsets (ages 3-14) has also been found to reduce children's pain and fear, while increasing parental satisfaction ^36^.

Administering the vaccine quickly is an easy and effective method to reduce pain ^37^. When multiple vaccines are administered in the same session, it is recommended to inject the less painful vaccines first and the more painful ones last. This approach may reduce the child's overall pain experience and help prevent the development of negative attitudes toward vaccination ^38^. Effective pain management plays a crucial role in gaining parental acceptance of medical procedures like vaccination and improving child cooperation ^39^. Healthcare professionals are more likely to achieve optimal analgesic effects when combining various non-pharmacological pain management strategies ^28^.

A systematic review recommends the following strategies for reducing vaccine-related pain: using the least painful commercial vaccine formulation, holding the child or infant during the procedure, gently stroking the child before and during the procedure, applying pressure near the injection site, and administering the intramuscular injection quickly without aspiration ^40^. It was observed that the methods used by participants to reduce children's pain and fear were consistent with the literature. In addition to traditional practices, the dissemination of scientifically proven methods can be facilitated through educational programs such as this one.

Midwives and nurses are generally the strongest advocates of immunization programs. It is well known that healthcare professionals' knowledge and attitudes about vaccines, their own previous vaccination experiences, and their intentions to recommend vaccines to their patients are significant determinants of patient vaccine uptake ^41^. However, vaccine hesitancy is also reported to be common among healthcare workers ^42^. Despite some pediatric nurses expressing intent to vaccinate their own children, nearly one-third were found to experience vaccine hesitancy, particularly with regard to human papillomavirus and varicella vaccines ^22^. Individuals who are vaccine-hesitant may refuse certain vaccines, accept some, or delay vaccination.

In addressing parental concerns about vaccination, the interaction and communication between healthcare personnel and parents are known to play a key role in reducing hesitations. There is a growing need to enhance the training of healthcare providers in combating vaccine hesitancy and countering widespread anti-vaccine myths. The most commonly used educational approaches in this context are the presumptive approach and motivational interviewing techniques ^21^^,^^43^^,^^44^.

A randomized controlled trial that assessed mothers' vaccine literacy and advocacy found that an educational intervention significantly increased vaccine advocacy and positive attitudes toward vaccination. It is recommended that healthcare training programs for professionals particularly in areas such as infection control and immunization be conducted regularly and continuously to update and refresh knowledge ^20^^,^^45^^-^^47^.

The advocacy role of healthcare professionals especially nurses and midwives in immunization programs is vital. They are not only implementers of vaccines but also serve as trusted sources of information and role models for the public. Nevertheless, even individuals with professional knowledge may experience vaccine hesitancy. This highlights the importance of healthcare professionals not only as technical implementers but also as effective communicators and advocates. Therefore, it is essential that vaccine education in health systems should not be limited to technical knowledge. It should also be enriched with content focused on communication skills and advocacy in order to strengthen the role of healthcare workers in promoting vaccine acceptance and combating hesitancy.

Although baseline vaccine advocacy scores were relatively high, the statistically significant increase observed after the training suggests that targeted vaccine advocacy education can further strengthen healthcare professionals' confidence, attitudes, and readiness to advocate for vaccination. Vaccination is widely recognized as one of the most effective and cost-efficient public health interventions aimed at improving child health. Its proven safety and efficacy help prevent numerous infectious diseases.

Vaccination practices contribute to herd immunity by reducing the spread of infectious diseases and save millions of lives annually. In this context, it is of great importance that nurses and midwives possess up-to-date knowledge on immunization, have confidence in their abilities, and develop effective communication skills. Healthcare professionals play a key role in promoting the acceptance of immunization practices and in building public trust in vaccines. The findings of this study provide evidence that short-term, structured in-service training interventions are effective in producing statistically measurable improvements in vaccine advocacy levels.

### Limitations and strengths

As the data were collected through a self-reported questionnaire, there may have been a social desirability bias, with participants potentially presenting themselves in a more favorable light. The attitudes and advocacy behaviors of midwives and nurses regarding vaccination are influenced by their cultural and social contexts, which may vary across countries or communities. Moreover, due to the limited number of studies using this scale in the literature, it is difficult to make comparisons across different groups or time periods.

In today's context, where vaccine hesitancy and opposition are increasing, there is a growing need for tools that specifically measure vaccine advocacy. This scale is significant in that it addresses a current public health concern. Unlike tools that measure general attitudes, it is uniquely designed to directly assess the advocacy role of healthcare professionals regarding vaccination.

The use of this focused and purpose-built vaccine advocacy scale represents a notable strength of the study.

The datasets used and/or analysed during the current study are available from the corresponding author on reasonable request.
